# A structure-based engineering approach to abrogate pre-existing antibody binding to biotherapeutics

**DOI:** 10.1371/journal.pone.0254944

**Published:** 2021-07-23

**Authors:** Joanne Lin, Stacey L. Lee, Anna M. Russell, Rong Fong Huang, Micheal A. Batt, Shawn S. Chang, Andrea Ferrante, Petra Verdino

**Affiliations:** Eli Lilly & Co, Lilly Biotechnology Center, San Diego, California, United States of America; National Research Council Canada, CANADA

## Abstract

Development of biotherapeutics is hampered by the inherent risk of immunogenicity, which requires extensive clinical assessment and possible re-engineering efforts for mitigation. The focus in the pre-clinical phase is to determine the likelihood of developing treatment-emergent anti-drug antibodies (TE-ADA) and presence of pre-existing ADA in drug-naïve individuals as risk-profiling strategies. Pre-existing ADAs are routinely identified during clinical immunogenicity assessment, but their origin and impact on drug safety and efficacy have not been fully elucidated. One specific class of pre-existing ADAs has been described, which targets neoepitopes of antibody fragments, including Fabs, VH, or VHH domains in isolation from their IgG context. With the increasing number of antibody fragments and other small binding scaffolds entering the clinic, a widely applicable method to mitigate pre-existing reactivity against these molecules is desirable. Here is described a structure-based engineering approach to abrogate pre-existing ADA reactivity to the C-terminal neoepitope of VH(H)s. On the basis of 3D structures, small modifications applicable to any VH(H) are devised that would not impact developability or antigen binding. *In-silico* B cell epitope mapping algorithms were used to rank the modified VHH variants by antigenicity; however, the limited discriminating capacity of the computational methods prompted an experimental evaluation of the engineered molecules. The results identified numerous modifications capable of reducing pre-existing ADA binding. The most efficient consisted of the addition of two proline residues at the VHH C-terminus, which led to no detectable pre-existing ADA reactivity while maintaining favorable developability characteristics. The method described, and the modifications identified thereby, may provide a broadly applicable solution to mitigate immunogenicity risk of antibody-fragments in the clinic and increase safety and efficacy of this promising new class of biotherapeutics.

## Introduction

Biotherapeutics are the most rapidly evolving drug class with monoclonal antibodies constituting the majority of biomolecules in development [1]. Recently, novel antibody-derived formats, including multi-specifics, antibody fragments, as well as other small binding scaffolds have entered the clinic, bringing new opportunities and challenges [2]. Small Ab fragments have gained considerable attention due to distinct properties from full-length antibodies; i.e., although these constructs maintain specific high-affinity binding, facile developability, and cost-effective manufacturing, altered tissue distribution, pharmacokinetics, and assembly into modular multi-specifics are possible. These features are especially attractive for applications where small size is advantageous, such as penetration of solid tumors or imaging applications [3]. In the clinic, the most successful small binding scaffolds tested to date are VHH single-domain antibodies with numerous molecules in various clinical stages [3] and Caplacizumab approved in 2018 for treatment of a rare blood disorder [4].

One key aspect of any protein biotherapeutic, in particular engineered non-naturally occurring molecules, is the inherent risk of immunogenicity. Any changes such as chemical modifications of side chains, fragmentation or aggregation can elicit unwanted adverse reactions and impact the clinical safety and efficacy profiles [5, 6]. In addition to treatment-induced immune responses, occasionally pre-existing ADAs are detected in drug-naïve individuals [7–9]. While the origin of pre-existing ADAs is poorly understood and the clinical impact varies case-by-case, it is recognized and established that pre-existing ADAs represent an additional challenge in the development of novel protein therapeutics [10]. One notable class of pre-existing ADAs identified are directed against antibody fragments, including the neo-epitopes in the hinge region in human Fab or (Fab’)_2_ fragments [11, 12] or a C-terminal neo-epitope exposed in VH or VHH domains outside the natural IgG context [7–9]. Recent evidence supports a role of pre-existing ADAs against antibody fragments in the host response to IgG cleavage by proteases that are prevalent in certain disorders [13]. Inflammation- and tumor-related as well as microbial proteases are capable of cleaving human IgG specifically in the hinge region [14, 15]. This might be a strategy of tumors and microbes to avoid immune-surveillance under the coating of dysfunctional F(ab’)2 (or similar) fragments. In turn, the human immune system may respond by the expansion of autoreactive B cells and production of antibodies that are specific for these novel exposed neo-epitopes [13]. Similar mechanisms could be envisioned to underly the generation of anti-VH(H) pre-existing ADA. Up to ~2/3 of healthy individuals have measurable levels of anti-hinge antibodies [15], and approximately 50% of healthy humans have anti-VH(H) antibodies [16]. In a clinical trial, infusion reactions consistent with cytokine release were observed and traced back to the cross-linking of monovalent VH domains by such pre-existing ADAs [16]. Interestingly, the addition of amino acids that are naturally present as part of the full-length antibody sequence significantly reduced frequency of pre-existing ADA binding [17].

In this work, the goal was to develop a structure-based approach to abrogate pre-existing ADAs binding to the C-terminal neoepitope of VH(H)s. By analyzing the atomic interactions at the VH(H) C-terminus and inducing structural changes, pre-existing ADA binding was disrupted while concomitantly reducing the risk of introducing new T cell epitopes. Linear and conformational B cell epitope predictions were evaluated in parallel with experimental characterization of identified molecular designs to significantly reduce pre-existing ADAs binding. This research demonstrates that the elimination of potential immunogenicity-related liabilities can be achieved through the iteration of structural analysis and *in-vitro* experimental validation, thus augmenting the poor discriminating capacity of *in-silico* B cell epitope prediction.

## Materials and methods

### Structure-based design of VHH variants

The RCSB PDB data base (rcsb.org; [18]) was mined for camelid VHH and autonomous human VH 3D X-ray structures. Approximately 300 total PDB entries were retrieved for VHH or VH domains alone or in complex with other proteins. The largest number of VHH PDB entries were derived from llama (Lama glama) (n = 120), alpaca (Vicugna pacos) (n = 82), and camel (Camelus dromedarius) (n = 62). Fourteen structures were retrieved for autonomous human VH domains (refinement resolution range of 1.5 to 2.8Å). Due to their large number, VHH structures were divided into 3 subsets by species and only high-resolution structures with refinement resolutions better than 2Å were included for further analysis. This filtering process resulted in 47 structures for llama VHHs, 41 for alpaca VHHs, and 57 for camel VHHs. The program MOE (Molecular Operating Environment (MOE), 2019.01; Chemical Computing Group ULC) was used to remove non-VH(H) proteins, solvent atoms and ligands from the PDB files. VHH or VH CDRs were annotated according Kabat’s CDR definitions [19]. CDR regions, as expected, displayed high sequence and structural variability, while superimpositions of only framework residues yielded RMSD values well below 1Å, indicating remarkable structure conservation. Both amino acid sequences and their structural arrangement in the C-terminal portion of VHs and VHHs were found to be highly conserved. This information was then used to design VHH variants with structural changes to abrogate pre-existing ADA binding to the VHH C-terminus.

### Homology modeling

Homology models of VHHs with unmodified C-terminus and various C-terminal designs to abrogate pre-existing ADA binding were generated using the protein modeler tool in the “Antibody Modeling” suite of the program MOE (Molecular Operating Environment (MOE), 2019.01; Chemical Computing Group ULC). Multiple VHH templates were generated from the pool of existing VHH structures from the RCSB PDB database (rcsb.org; [18]). Preferred templates for CDR loops based on amino acid identity and the number of amino acids in each of corresponding CDR loops were selected. Constructed models were protonated according to pKa predictions at pH 7.4, then the Amber10 ETH force field [20] was assigned. This permitted full minimization of the VHH homology models in implicit solvent simulated by a Generalized Born Solvation model [21]. Graphical representations were generated with Pymol (The PyMOL Molecular Graphics System, Version 2.0 Schrödinger, LLC).

### B cell epitope predictions

*In-silico* identification of linear B cell epitopes was carried out with Ellipro and Bepipred, and conformational epitopes were predicted with Ellipro, both available through the IEDB Analysis Resources. BepiPred predicts the location of linear B-cell epitopes using a combination of a hidden Markov model and a propensity scale method, resulting in a score [22]. The residues with scores above the threshold are predicted to be part of an epitope. The threshold was varied in the 0.90–1.15 range to assess changes in antigenicity associated with the different cutpoints. ElliPro predicts linear and discontinuous antibody epitopes based on a protein antigen’s 3D structure [23]. ElliPro accepts as an input protein structure in PDB format. The respective PDB files from the models generated as explained in the previous paragraph were used as the input for ElliPro analysis.

### Cloning, test expression, and SDS-PAGE analysis

A VHH sequence was synthesized (IDT, Coralville, IA) with a 6X His-tag fused to the N-terminus with a three amino acid linker (GGS). Nineteen variations of the His-tagged VHH sequence, including various amino acid additions, deletions, or substitutions, were designed at the C-terminus or framework 1 and were also synthesized (IDT, Coralville, IA) (Table 1). The twenty sequences were cloned into a GS-containing expression plasmid backbone (pEE12.4-based plasmid) with the viral CMV promoter. The VHH sequences were fused in frame with the coding sequence of a signal peptide sequence, METDTLLLWVLLLWVPGSTG, to enhance secretion of the VHH variants into the tissue culture medium.

**Table 1:** Structure-based designs to abrogate pre-existing ADA binding to VHH C-terminus.

Design Rational	VHH Variant	Amino Acid Change
Unmodified	MC6.1	none
Design from Cordy *et al*. 2015	MC6.40	Ala114
*Change overall molecular structure/surface*		
Replace hydrophobic with negatively charged side-chain	MC6.41	Leu11Glu
Add bulky residues with low likelihood of HLA binding	MC6.42	Pro114
	MC6.43	Pro114, Pro115
	MC6.44	Pro114, Pro115, Pro116
	MC6.45	Ser113Pro
Disrupt Ser112 main-chain H-bond to Val12 and side-chain H-bond to Gln13	MC6.46	Ser112Pro, desSer113
Remove side chains (w/ or w/o addition of prolines)	MC6.47	Ser113Ala
	MC6.48	Ser113Ala, Pro114
	MC6.49	Ser112Gly, Ser113Gly
	MC6.50	Ser112Gly, desSer113
	MC6.51	Ser112Gly, Ser113Pro
	MC6.52	Ser113Gly, Pro114
*Generate additional hydrogen bonds*		
Bidentate H-bond Gln13 with Ser113Gln	MC6.53	Ser113Gln
Bidentate H-bond Gln13 with Ser113Gln	MC6.54	Ser113Gln, Pro114
H-bond Thr110 to Leu11Ser (llama residue)	MC6.55	Leu11Ser
H-bond Thr110 to Leu11Ser (llama residue)	MC6.56	Leu11Ser, Ser112Gln, desSer113
H-bond Thr110 to Leu11Gln	MC6.57	Leu11Gln
Bidentate H-bond Leu11Gln to Ser113Gln	MC6.58	Leu11Gln, Ser113Gln

The VHH variants were recombinantly expressed from 2mL cultures of transiently transfected CHOK1 suspension cells using a PEI-based method, as previously described [24, 25]. At the end of the incubation period, cells were removed by low-speed centrifugation and supernatants containing the VHH proteins were harvested for subsequent SDS-PAGE analysis. The supernatants, 10μL, for all 20 VHH variants were analyzed on a 12% polyacrylamide SDS-PAGE gel (BioRad, #345–0118), stained by Simply Blue Safestain (Invitrogen, #LC6060) and destained overnight in distilled water. Reduced samples were mixed 1:20 (v/v) with reducing agent (BioRad, #161–0792), 1:4 (v/v) with 4x loading dye (BioRad, #161–0791) and heated at 95 ^o^C for 5 min before loading onto the gel. Non-reduced samples were mixed with 4x loading dye (BioRad, #161–0791) before loading onto the gel. Images of the destained gels were acquired using a UVP EpiChemi3 Darkroom imager with white light and UVP Visionworks LS software. Image editing was limited to cropping and labeling only.

### Large-scale expression and purification

The VHH variants were recombinantly expressed from transient transfections of CHOK1 suspension cells using a PEI-based method, as previously described [24, 25]. At the end of the incubation period, cells were removed by low-speed centrifugation and supernatants containing the VHH proteins were harvested for subsequent 2-step purification.

The initial capture step required that a 1-mL HiTrap Ni-NTA FF column (GE Healthcare) be equilibrated with buffer A (20mM Tris-HCl pH 8.0, 0.5M NaCl) at 1mL/min flowrate. Cell culture supernatants were loaded onto the column at 1mL/min followed by a wash step with 5 column volumes of buffer A. Then, a step gradient to 100% buffer B (20mM Tris-HCl pH 8.0, 0.5M NaCl, 250mM imidazole) over 10 column volumes were applied. Elution fractions containing the VHH variants were collected, pooled, and concentrated to 2mL with a Vivaspin 6, 5K MWCO concentrator. Subsequent purification was performed on a 100-mL Sepax SRT 10C SEC300 column pre-equilibrated with 1x PBS pH7.4 at 3mL/min. The concentrated Ni-NTA pools were loaded and isocratically eluted at 3mL/min over 1-column volume. Fractions containing the VHH variants were collected, concentrated, and the protein concentration was determined by measuring the absorption at 280nm on a Nanodrop 200 Spectrophotometer.

### Thermal stability assay

Thermostability of the VHH variants was monitored using a Thermal Denaturation Fluorimetry (TDF) thermofluor assay run on a Roche LightCycler 480II PCR machine with SYPRO Orange dye (5000× concentrate, Invitrogen S6651). Excitation and emissions filters were set at 465nm and 580nm, respectively, and the temperature was ramped continuously from 25°C to 95°C at a rate of 0.1°C/second. Final assay conditions contained 10X SYPRO dye with VHH variants at 0.3 mg/mL or 0.6 mg/mL in either PBS pH 7.4 (Corning 21-040-CV) or 25 mM Tris pH 6.6, 7.5 mM citric acid. The experiment was performed by diluting each variant to 2× of the final concentration and mixing it in equal parts with 20× SYPRO Orange dye in the appropriate buffer to reach a final volume of 30 μL per sample. The mixture was dispensed as 6 μL aliquots in triplicate into a 384-multiwell assay plate (Roche 04-729-749-001). The T_m_ values for each variant was determined using the Thermal Shift Analysis Software (Roche) by the first derivative method. The analysis software smoothed the raw fluorescence data, and the T_m_ was collected by determining the temperature where the upward slope of fluorescence vs. temperature was maximal (inflection point). The mean T_m_ value was calculated and recorded for each triplicate set for each VHH and used for thermostability comparisons.

### Pre-existing ADA detection ELISA assay

Presence of anti-VHH pre-existing ADA in healthy donor sera was identified with an indirect ELISA. Fifty μL of each VHH variant (8 μg/mL in casein blocker) were captured via their His-tag onto Nickel Coated Plates (Pierce Cat. #15442). Plates were incubated at room temperature for 1h, after which 50 μL of a 1:128 dilution of human serum from healthy naïve donors into PBS were added. Sera were commercially acquired from the San Diego Blood Bank (approx. 50:50 males:females and >17y/o). Presence of anti-VHH immunoglobulins was detected with HRP-labeled anti-Fc gamma antibodies from Jackson ImmunoResearch Lab. (Cat. No #109-035-09). Fifty μL of 1-Step Ultra TMB (Thermo Fisher) was added to each well and the reaction was stopped after 10min with 50 μL of 2N H_2_SO_4_. Absorbance at 450nm was read on a Molecular Devices Spectramax plate reader.

### SPR affinity measurements of VHH variants binding to human serum albumin

Binding of VHH variants MC6.1, MC6.40, MC6.43, MC6.51, MC6.52, MC6.56 and MC6.58 to human serum albumin was measured on a Biacore 8K instrument. Human serum albumin was obtained from Sigma Aldrich (St. Louis, MO) (Cat. No. A8763). Immobilization of human serum albumin to a Series S Sensor Chip CM5 surface was performed according to manufacturer’s instructions (amine coupling kit BR-1000-50, Cytiva). Briefly, carboxyl groups on the sensor chip surfaces (flow cell 1 and 2) were activated by injecting 70 μl of mixture containing 75 mg/ml 1-Ethyl-3-(3-dimethylaminopropyl) carbodiimide hydrochloride (EDC), and 11.5 mg/ml N-Hydroxysuccinimide (NHS), at 10 μl/min. Human serum albumin was diluted in 10 mM sodium acetate pH 4.0 (BR-1003-49) to 0.8 μg/ml, then injected over the activated chip surfaces (flow cell 2, channel 1 to 8) at 10 μl/min for 100 seconds. Human serum albumin was covalently immobilized through free amines onto the carboxymethyl dextran-coated sensor chip targeting a surface density of approximately 30 resonance units (RU). Excess reactive groups on the surfaces (flow cell 1 and 2) were deactivated by injecting 70 μl of 1 M Ethanolamine hydrochloride-NaOH pH 8.5 at 10 μl/min.

VHH variants were diluted in HBS-EP+ buffer (10 mM HEPES pH 7.6, 150 mM NaCl, 3 mM EDTA, 0.05% Polysorbate 20) at concentrations of: 1000, 333.3, 111.1, 37.04, 12.35, 4.12, 1.37, 0.457, 0.152, 0.051, and 0.017 nM. One hundred-eighty μl of sample was individually injected sequentially across the immobilized serum albumin surface and let dissociate for 600 seconds at 60 μl/min flow rate at 25°C. The surface was regenerated by injecting 10 mM glycine-HCl pH 1.5 (BR-1003-54) at 60 μl/min for 100 seconds. The resulting sensorgrams were analyzed using Biacore 8K Insight Evaluation Software (version 2.0.15.12933) applying global fitting of a 1:1 binding model to calculate the association rate (ka), dissociation rate (kd), and equilibrium dissociation constant (K_D_).

### Plasma stability assay

VHH variants MC6.40, MC6.43, MC6.51 and MC6.56 were tested for their stability in mouse plasma. Previously frozen mouse plasma (-80°C) was thawed and filtered using 0.22 μm Ultrafree MC centrifugal filters (Sigma Aldrich) for 5 min at 10,000 g. For each VHH variant, 125 μg were transferred to a 1.8 mL Eppendorf tube. The total volume was brought to 1 mL with the filtered mouse plasma, achieving a greater than 10-fold dilution. Samples were vortexed and then set on a thermomixer at 37°C and 350 rpm. At each time point (1, 6, 24, 48 h), a 200 μL (approx. 25 μg) sample was removed and the VHH variants were extracted using HISPurTM Ni-NTA spin columns (ThermoScientific). Spin columns were equilibrated to room temperature and the bottom tabs were removed. Each column was placed into an Eppendorf tube and centrifugated for 2 min at 700 g to remove storage buffer. Equilibration Buffer, 400 μL, was added and the column was placed in a new Eppendorf tube. After a 2 min wait, the column was centrifugated for 2 min at 700 g to remove Equilibration Buffer. The bottom plug was placed back on the column and 200 uL of mouse plasma stability sample were added to 200 μL of Equilibration Buffer in each spin column. The spin column was taped into a new Eppendorf tube and mixed on an orbital mixer at room temperature for 30 min. The bottom plug was removed, and the column centrifuged for 2 min at 700 g. The column was placed into a new Eppendorf tube, washed with 400 μL of Wash Buffer and centrifuged for 2 min at 700 g. The flow through was kept and the process repeated 2 more times. The column was placed in a new Eppendorf tube and 200 μL of Elution Buffer were added. The column was centrifuged for 2 min at 700 g and the process repeated 2 more times. All 3 elutions were combined and the volume reduced by Speed Vac to approximately 400 μL. The column was washed with 400 μL of Elution Buffer and centrifuged for 2 min at 700 g. The combined eluates were buffer-exchanged with Zeba Spin columns (7K MWCO) using 50 mM Tris-HCl pH 7.5 and dried down to 50–100 μL with a speed vac. Samples from the various timepoints were then analyzed by mass spectrometry to determine integrity of the VHH variants.

## Results and discussion

### Structure-based design of VHH variants

**Fig 1:**
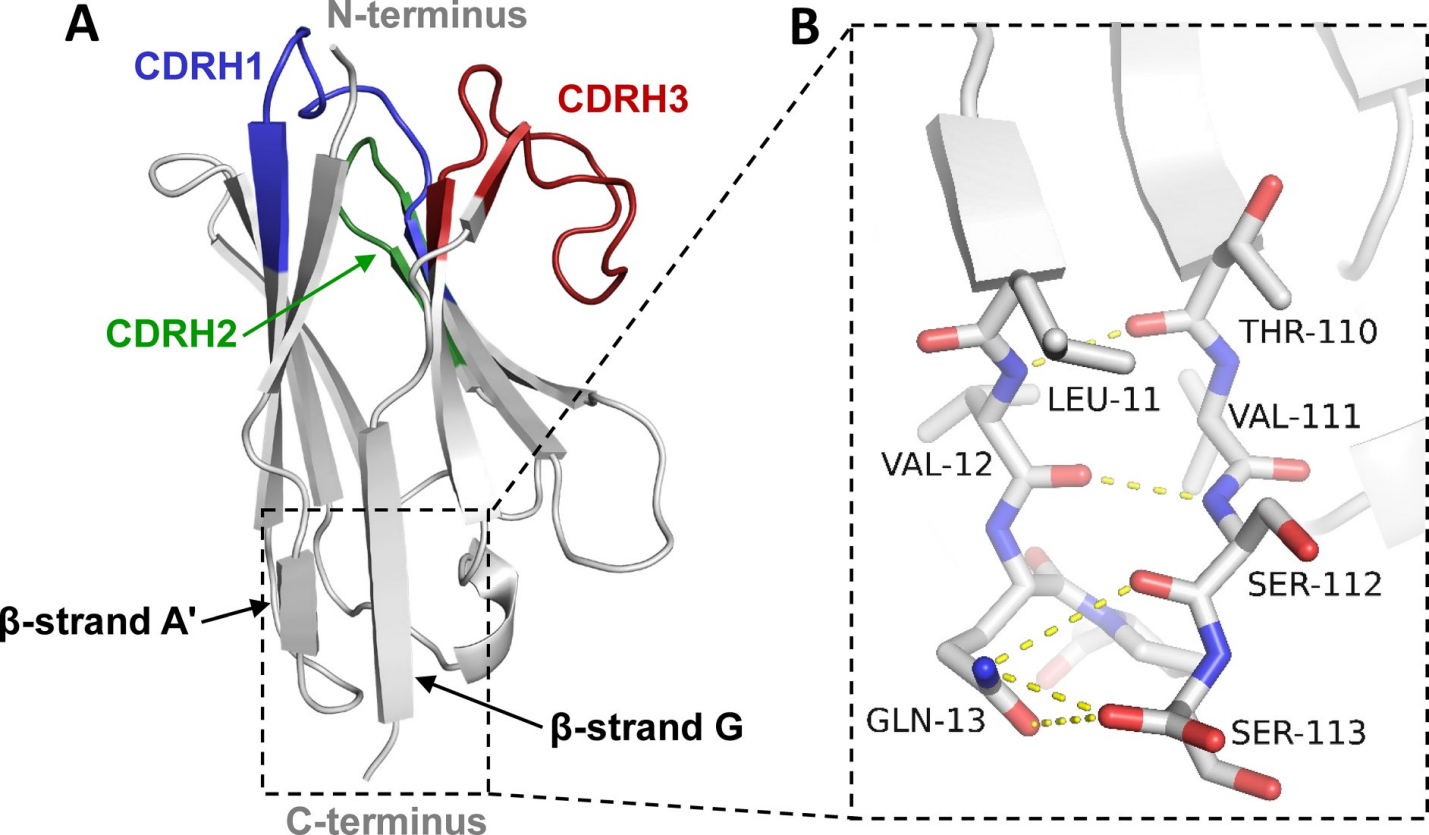
Structural analysis of the VHH C-terminus to aid the design of variants with abrogated pre-existing ADA binding. (A) Cartoon representation of a representative VHH structure. N- and C-termini are indicated and the three CDRs are color coded in blue (CDRH1), green (CDRH2), and red (CDRH3). (B) Close-up view of the residues constituting the VHH C-terminus and their structural interactions with surrounding residues. Hydrogen-binding interactions are shown as yellow dashes. Main chain hydrogen bonds are formed from Thr110 to Leu11 and Ser112 to Val12. In addition, the side chain of Gln13 forms a bidentate hydrogen bond to the C-terminal carboxy group of Ser113 and an additional hydrogen bond to the main chain carbonyl of Ser112. Furthermore, there are hydrophobic packing interactions between the side chains of Val111 and Val12 as well as Thr110 and Leu11.

Pre-existing antibodies, like antibodies in general, recognize both conformational as well as linear epitopes. Thus, to abrogate pre-existing ADA binding to the C-terminal neoepitope of isolated VH and VHH domains, a structure-based approach was utilized. First, a total of 159 crystal structures of VHHs and autonomous human VH domains from the RCSB PDB database (rcsb.org; [18]) were compared. It was observed that their overall structures as well as the amino acid sequences and structural arrangements in their C-terminal portion are highly conserved (S1-S5 Figs in S1 File). A network of molecular interactions occurs at the C-terminus: Residues Thr110, Val111, and Ser112 are part of a β-sheet composed of the G and A’ β-strands of the IgV domain, which also includes residues Leu11 and Val12 (Fig 1A and 1B). The C-terminal Ser113 forms two main chain H-bonds to the side chain of Gln13. In addition, the structural arrangement is stabilized by hydrophobic packing interactions between side chains of Leu11 and Val12 with Thr110 and Val111, respectively (Fig 1B).

For potential modifications that would abrogate pre-existing ADA binding to the VH(H) C-terminus, the following constraints were considered:

1. Structural changes should remain localized to the VHH C-terminus to avoid negative impact on overall structure. This enables the same pre-existing ADA designs across VHs and VHHs without impacting antigen binding or destabilizing the molecule.
2. Changes should have a low likelihood of inducing *de novo* immunogenicity. Therefore, preferably proline and glycine residues were utilized in the pre-existing ADA designs as those have been reported to confer low complex stability in HLA-II-binding peptides [26].

Based on those constraints, a total of 18 new VHH variants were generated, which featured the addition of extra amino acids, disruption of H-bonds to reorient amino acid side chains, changes of main-chain conformation, and the removal of side chains (Table 1). Some designs were also included to interrogate the potential contribution of Leu11 to the C-terminal pre-existing ADA epitope. Leu11 is known for its close interactions with residues in the antibody constant region [27]. In isolated VH and VHHs, the Leu11 side chain becomes highly solvent-exposed, and it was hypothesized that this new hydrophobic surface might contribute to the pre-existing ADA epitope.

### *In-silico* predictions of B cell epitopes at the C-termini of the VHH variants

**Fig 2:**
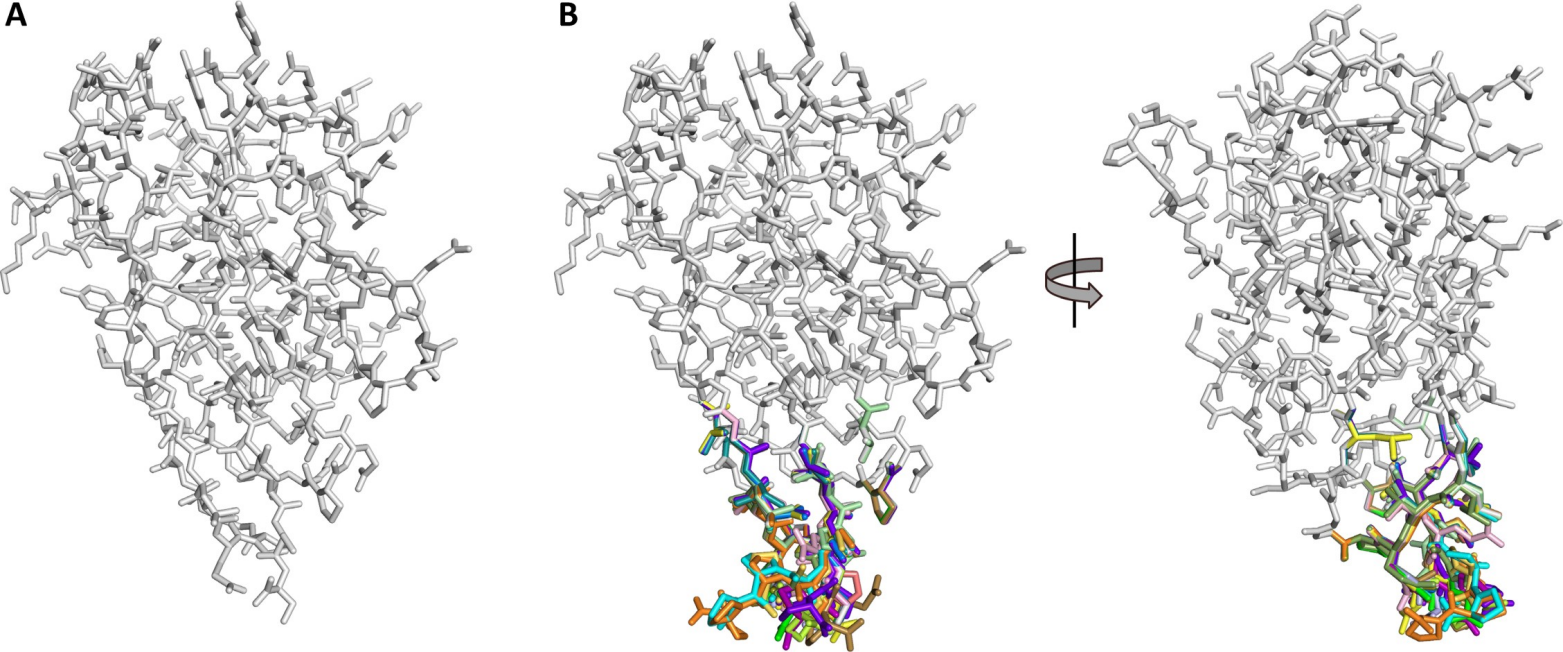
Structure models of various VHH variants with pre-existing ADA designs. (A) Computational model of the non-modified VHH MC6.1 in a ribbon representation. (B) Structure superimposition of computational models of the non-modified VHH MC6.1 (grey) and the VHH variants with various C-terminal pre-existing ADA designs (color). The C-terminal amino acid substitutions, truncations and additions result in a wide variety of conformational arrangements, all of which are limited to the C-terminal portion of the molecule and do not appear to impact the overall VHH structural integrity.

Homology models of VHHs of unmodified C-terminus (Fig 2A) and various C-terminal designs (Fig 2B) were generated using the protein modeler tool in the “Antibody Modeling” suite of the program MOE. VHH templates were generated from the pool of existing VHH structures from the RCSB PDB data base. The C-terminal designs were implemented and each VHH variant was subjected to energy minimization. Superimposition of the homology models showed that structural perturbations indeed remained limited to the VHH C-terminus (Fig 2B). In addition, the various designs represented a wide variety of distinct structural conformations as desired.

To predict whether the modified C-termini would constitute less likely targets for pre-existing antibodies as compared to the unmodified sequence, two *in-silico* B cell epitope mapping tools were used. Because antibodies can recognize both linear and conformational epitopes, both Ellipro and BepiPred were utilized to assess both scenarios. The results of these analyses are reported in Tables 2 and 3, respectively.

**Table 2:** Ellipro-based prediction of conformational B cell epitopes.

VHH Variant	Conformational Epitope Sequence	Conformational Score	Linear Epitope Sequence	Linear Score
MC6.1	G9, G10, L11, V12, Q13, P14, G15, G16, S17, A61, D62, S63, K65, G66, R67, AN84, S85, L86, R87, P88, E89, T91, T122, S124, S125	0.67	N/A	0
MC6.40	G9, G10, L11, V12, Q13, P14, G15, G16, S17, A61, D62, S63, V64, K65, G66, R67, N84, S85, L86, R87, P88, E89, T91, T122, S124, S125, A126	0.644	TVSSA	0.798
MC6.41	G9, G10, E11, V12, Q13, P14, G15, G16, S17, A61, D62, S63, K65, G66, R67, N84, S85, L86, R87, P88, E89, T91, T122, V123, S124, S125	0.663	TVSS	0.774
MC6.42	L11, V12, Q13, P14, G15, G16, S17, A61, D62, S63, K65, G66, R67, N84, S85, L86, R87, P88, E89, T91, T122, S124, S125, P126	0.686	TVSSP	0.808
MC6.43	L11, V12, Q13, P14, G15, G16, S17, A61, D62, S63, K65, G66, R67, N84, S85, L86, R87, P88, E89, T91, T122, S124, S125, P126, P127	0.679	TVSSPP	0.801
MC6.44	L11, V12, Q13, P14, G15, G16, S17, A61, D62, S63, V64, K65, G66, R67, N84, S85, L86, R87, P88, E89, T91, T122, S124, S125, P126, P127, P128	0.659	TVSSPPP	0.81
MC6.45	G9, G10, L11, V12, Q13, P14, G15, G16, S17, A61, D62, S63, K65, G66, R67, N84, S85, L86, R87, P88, E89, T91, T122, V123, S124, P125	0.666	N/A	0
MC6.46	V12, Q13, P14, G15, G16, S17, A61, D62, S63, K65, G66, R67, N84, S85, L86, R87, P88, E89, V123, P124; G9, G10, L11, T91, T122	0.675	N/A	0
MC6.47	G9, G10, L11, V12, Q13, P14, G15, G16, S17, A61, D62, S63, K65, G66, R67, N84, S85, L86, R87, P88, E89, T91, T122, V123, S124, A125	0.665	N/A	0
MC6.48	G9, G10, L11, V12, Q13, P14, G15, G16, S17, A61, D62, S63, K65, G66, R67, N84, S85, L86, R87, P88, E89, T91, T122, S124, A125, P126	0.665	TVSAP	0.802
MC6.49	G9, G10, L11, V12, Q13, P14, G15, G16, S17, A61, D62, S63, K65, G66, R67, N84, S85, L86, R87, P88, E89, T91, T122, G124, G125	0.668	N/A	0
MC6.50	G9, G10, L11, V12, Q13, P14, G15, G16, S17, A61, D62, S63, K65, G66, R67, N84, S85, L86, R87, P88, E89, T91, T122, V123, G124	0.673	N/A	0
MC6.51	L11, V12, Q13, P14, G15, G16, S17, A61, D62, S63, K65, G66, R67, N84, S85, L86, R87, P88, E89, T91, T122, G124, S125, P126	0.681	TVGSP	0.8
MC6.52	G9, G10, L11, V12, Q13, P14, G15, G16, S17, A61, D62, S63, K65, G66, R67, N84, S85, L86, R87, P88, E89, T91, T122, S124, G125, P126	0.666	TVSGP	0.802
MC6.53	G9, G10, L11, V12, Q13, P14, G15, G16, S17, A61, D62, S63, K65, G66, R67, N84, S85, L86, R87, P88, E89, T91, T122, V123, S124, Q125	0.664	N/A	0
MC6.54	G9, G10, L11, V12, Q13, P14, G15, G16, S17, A61, D62, S63, K65, G66, R67, N84, S85, L86, R87, P88, E89, T91, T122, S124, Q125, P126	0.667	TVSQP	0.803
MC6.55	G9, G10, S11, V12, Q13, P14, G15, G16, S17, A61, D62, S63, K65, G66, R67, N84, S85, L86, R87, P88, E89, T91, T122, S124, S125	0.671	N/A	0
MC6.56	G9, G10, S11, V12, Q13, P14, G15, G16, S17, A61, D62, S63, V64, K65, G66, R67, N84, S85, L86, R87, P88, E89, T91, T122, V123, Q124	0.644	N/A	0
MC6.57	G9, G10, Q11, V12, Q13, P14, G15, G16, S17, A61, D62, S63, K65, G66, R67, N84, S85, L86, R87, P88, E89, T91, T122, V123, S124, S125	0.665	TVSS	0.774
MC6.58	G9, G10, Q11, V12, Q13, P14, G15, G16, S17, A61, D62, S63, K65, G66, R67, N84, S85, L86, R87, P88, E89, T91, T122, V123, S124, Q125	0.66	TVSQ	0.764

**Table 3:** BepiPred-based prediction of linear B cell epitopes.

VHH Variant	Start	End	Peptide	Length
All except MC6.41, MC6.55, MC6.56, MC6.57, MC6.58	10	15	GLVQPG	6
All	19	26	RLSCAASG	8
All	31	37	STAVAWF	7
MC6.41, MC6.56	56	63	DITYYADS	8
MC6.41, MC6.55, MC6.56, MC6.57, MC6.58	78	83	TLYLQM	6
All	91	99/101	TAVYYCAVR(PG)	9/11

ElliPro predicts linear and discontinuous antibody epitopes based on a 3D structure of an antigen, which can be loaded as a PDB file [23]. There was no significant difference between unmodified VHH sequence with all the VHH variants in the likelihood that a cluster of residues containing the C-terminus would constitute a conformational epitope. Instead, this prediction indicated that 11 C-terminal designs would introduce a linear epitope that is not otherwise present in the parental sequence or the other seven variants.

BepiPred utilizes a Hidden Markov Model trained using a data set derived from the database Antijen and the Pellequer data set and combined with Parker’s hydrophilicity scale to predict the location of linear B-cell epitopes [22]. The residues with scores above the threshold are predicted to be part of an epitope. A threshold of 0.90 yields a sensitivity of 0.25 and a specificity of 0.91. Using this cutpoint, all the sequences would contain a linear epitope at the C-terminus. On the contrary, adoption of a higher threshold, such as 1.15, would decrease sensitivity (~0.18) and increase specificity (~0.93). With the higher threshold, none of the predicted linear B cell epitopes would comprise the C-terminal residues, including the parental VHH. Collectively, these results indicate that the ability to discriminate variants on the basis of the *in-silico* predicted reactivity to pre-existing antibodies is weak, and an experimental assessment strategy is required to rank these molecules more accurately.

### Generation and characterization of VHH variant proteins

**Fig 3:**
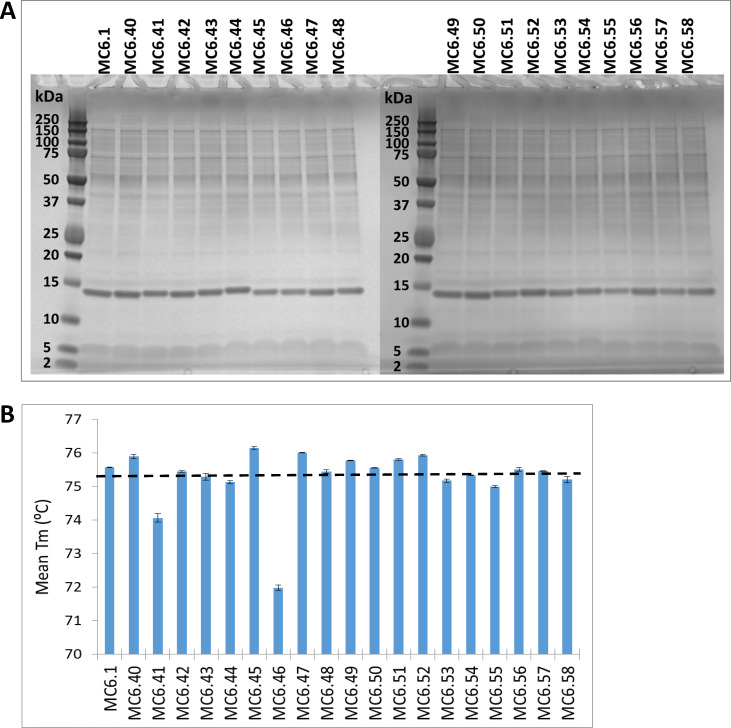
Expression and thermal stability of VHH variants. (A) Expression of VHH variants in transient CHO cell culture supernatants visualized by reducing SDS-PAGE. 10μl of supernatant were loaded into each lane. Molecular weight markers are in the further leftmost lanes on both gels. MC6.1 is the unmodified VHH, MC6.40 to MC6.58 are the various VHHs with pre-existing ADA designs. All VHH variants expressed comparably to the unmodified VHH MC6.1. (B) Mean melting temperature (Tm) for each VHH variant in a thermal shift assay. The unmodified VHH MC6.1 and most variants exhibit Tms of approx. 75–76°C (mean Tm of 75.3°C indicated by a black dashed line) except for MC6.41 and M6.46 which feature 1.5- and 3.5°C-reduced thermal stability.

Twenty variants of a human serum albumin-binding VHH were cloned for recombinant expression in mammalian cells. This included the humanized VHH (MC6.1) with an unmodified C-terminus; the same VHH with a C-terminal alanine addition that had been previously found to reduce pre-existing ADA binding [17] (MC6.40); and 18 new structure-based designs (MC6.41-MC6.58). It should be noted that a N-terminal His-tag was included in all constructs to facilitate purification and evaluation in the pre-existing ADA assay. Small-scale test expressions demonstrated comparable expression levels for all 20 VHH variants (Fig 3A), indicating that the designs did not negatively impact intracellular folding or stability in the cell culture. All 20 VHH variants were then expressed at large scale and purified to homogeneity using a 2-step purification process. Final purification yields of all 20 VHH variants were comparable (S6 Table in S1 File).

To ensure the pre-existing ADA designs did not induce any major structure disruptions, the thermal stability of the VHH variants was evaluated in a thermofluor assay (Fig 3B). All, but two VHH variants exhibited Tm values of 75–76°C, comparable to the unmodified VHH MC6.1 (mean Tm of 75.3°C ± 0.88°C for all VHHs). Interestingly, MC6.41 and MC6.46 had 1.5°C and 3.5°C reduced thermal stability, respectively. The statistically significant thermal stability reduction of MC6.46 (Ser112Pro, desSer113) likely results from disruption of the beta-sheet secondary structure due to changes in the main-chain conformation and hydrogen bonding from the Ser112Pro substitution. This hypothesis is also corroborated by the fact that MC6.50, which is identical to MC6.46 with exception of Ser112Gly versus Ser112Pro, does not have a reduced Tm.

In addition, selected VHH variants (MC6.1, MC6.40, MC6.43, MC6.51, MC6.52, MC6.56, and MC6.58) were tested for retained antigen binding. SPR-based affinity measurements showed comparable human serum albumin binding to any of the six VHH variants and the unmodified MC6.1 VHH (*K*_D_ ranging between 9.9E-12 and 2.7E-11 M) (Table 4 and S7 Fig in S1 File).

**Table 4:** SPR kinetic parameters of MC6.1 and VHH variants binding to human serum albumin.

VHH variant	k_a_ (1/Ms)	k_d_ (1/s)	K_D_ (M)	R_max_ (RU)	^χ2^
MC6.1	3.0E+06	6.6E-05	2.2E-11	7.8	3.06E-02
MC6.40	3.0E+06	8.1E-05	2.7E-11	7.8	1.16E-02
MC6.43	2.9E+06	7.9E-05	2.7E-11	7.9	1.22E-02
MC6.51	5.6E+06	5.5E-05	9.9E-12	8.5	3.91E-02
MC6.52	3.6E+06	6.8E-05	1.9E-11	8.3	1.36E-02
MC6.56	3.7E+06	6.7E-05	1.8E-11	8.4	1.26E-02
MC6.58	3.4E+06	8.2E-05	2.4E-11	7.1	1.27E-02

k_a_: association rate; k_d_: dissociation rate; K_D_: equilibrium dissociation constant; R_max_: analyte binding capacity of the surface; χ^2^: chi square

To verify that the structure-based designs would not be subjected to proteolytic cleavage in biological matrices, selected VHH variants (MC6.40, MC6.43, MC6.51 and MC6.56) were evaluated in a plasma stability assay. Up to the final 48h timepoint, all 4 VHH variants proved to be stable at 37°C without any detectable modifications in the C-terminal regions as monitored by mass spectrometry.

### Evaluation of pre-existing ADA binding to VHH variants

**Fig 4:**
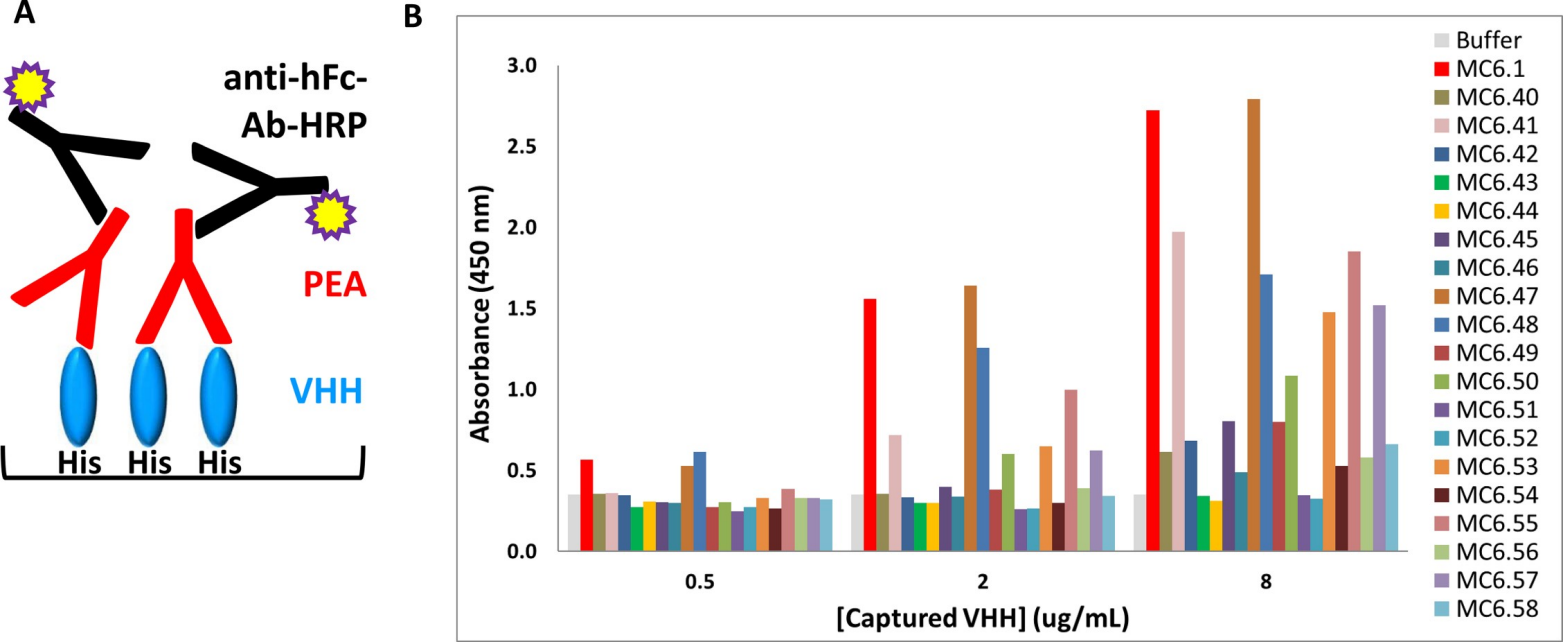
Screening ELISAs to identify the designs that most effectively abrogate pre-existing ADA binding. (A) Schematic representation of the ELISA assay used to evaluate pre-existing ADA binding to the VHH variants. N-terminal His-tagged VHH variants (blue) are captured on a Ni-NTA plate. The plate is then incubated with either a pool of or with individual human donor serums. Pre-existing ADA that recognize and bind to the VHH variants (red) are detected with an HRP-conjugated anti-human Fc Ab (black). (B) Binding of pre-existing ADA from a pool of sera of 10 naïve human individuals to VHH variants at 3 capture concentrations (0.5, 2, 8ug/ml). Different VHH variants have various levels of pre-existing ADA binding ranging from high for the unmodified MC6.1 and MC6.47 to low levels of pre-existing ADA binding (MC6.43, 44, 51, 52) comparable to the buffer control (no VHH captured).

All 20 VHH variants were evaluated against a pool of sera of 10 healthy human donors using an ELISA assay (Fig 4A). In the assay, the VHH with an unmodified C-terminus, MC6.1, showed a high level of pre-existing ADA binding (Fig 4B). The addition of a single alanine residue Ala114 in MC6.40, as previously described [17], led to a significant reduction of pre-existing ADA reactivity and validated the assay setup. MC6.47 (Ser113Ala) had comparable reactivity as MC6.1, indicating that the conservative serine to alanine modification of the C-terminal residue had no impact on recognition by pre-existing ADA. In contrast, a change to glutamine, MC6.53, did reduce pre-existing ADA reactivity to the same extent. Modifications of the Leu11 side chain, MC6.41 (Leu11Glu), MC6.55 (Leu11Ser), MC6.57 (Leu11Gln), led to minor reductions of pre-existing ADA binding, demonstrating that solvent exposure of the hydrophobic side chain is not a major contributor to the C-terminal pre-existing ADA epitope of isolated VH and VHH domains.

Removal of side chains of the C-terminal residues did show some promise, depending on the applied change. Interestingly, while combining a Ser113Ala change with the addition of an extra proline at residue 114 (MC6.48) had only a modest impact on pre-existing ADA reactivity, the same design using a Ser113Gly change (MC6.52) lowered pre-existing ADA reactivity to buffer background levels. Consistently, the promising designs were changes to or additions of proline residues, i.e., adding a single proline at position 114 (MC6.42) reduced signals from nearly 8-fold to 2-fold over buffer control while addition of two or three prolines (MC6.43 and MC6.44) lowered pre-existing ADA reactivity to the buffer control.

**Fig 5:**
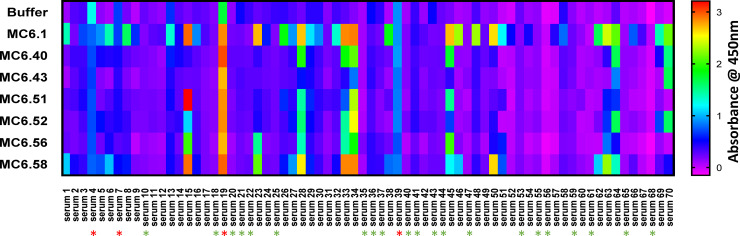
Pre-existing ADA binding from individual sera of 70 human donors to the unmodified VHH MC6.1 and six VHH variants. Heat map representation of the signals in pre-existing ADA binding ELISAs for six VHH variants screened against 70 individual sera from naïve donors. The selected six variants represent different design rationales that had low levels of pre-existing ADA binding in a primary screen against pooled sera. A wide variety of pre-existing ADA binding responses are observed between the different sera. Sera with significant adherence to the non-coated plate are indicated by red asterisks, while sera that exhibit no apparent pre-existing ADA reactivity are marked with green asterisks. Approximately 55% of all donors have pre-existing ADA reactivity against the unmodified MC6.1 VHH.

Based on these findings, the most promising VHH variants from each design group were selected for further evaluation against 70 individual human sera, namely: MC6.43, MC6.51, MC6.52, MC6.56, MC6.58 as well as Cordy’s design [17] MC6.40 and the unmodified MC6.1. A wide variety of pre-existing ADA binding responses was surprisingly observed between different sera (Fig 5). Some sera exhibited significant adherence even to a non-coated plate (i.e., serum 4, 7, 19, or 39). Twenty-one out of 70 sera (14%) had no apparent pre-existing ADA reactivity (e.g., serum 10, 18, 35, 40, 47, 56, 68). Overall, approximately 55% (38–42 out of 70, depending on cut-off) of all donors exhibited pre-existing ADA against the unmodified MC6.1 VHH. This is consistent with prior reports [16] of approximately 50% of healthy humans having anti-VH(H) antibodies. Despite a wide variety of quantitative pre-existing ADA reactivities from donor to donor, it was interesting to note that some sera responded specifically to some modifications while others did not; e.g., serum 23 had strong pre-existing ADA reactivity to MC6.1, MC6.56 and MC6.58, while serum 50 responded strongly to MC6.1 and MC6.58 and serum 48 only showed pre-existing ADA binding to MC6.1.

**Fig 6:**
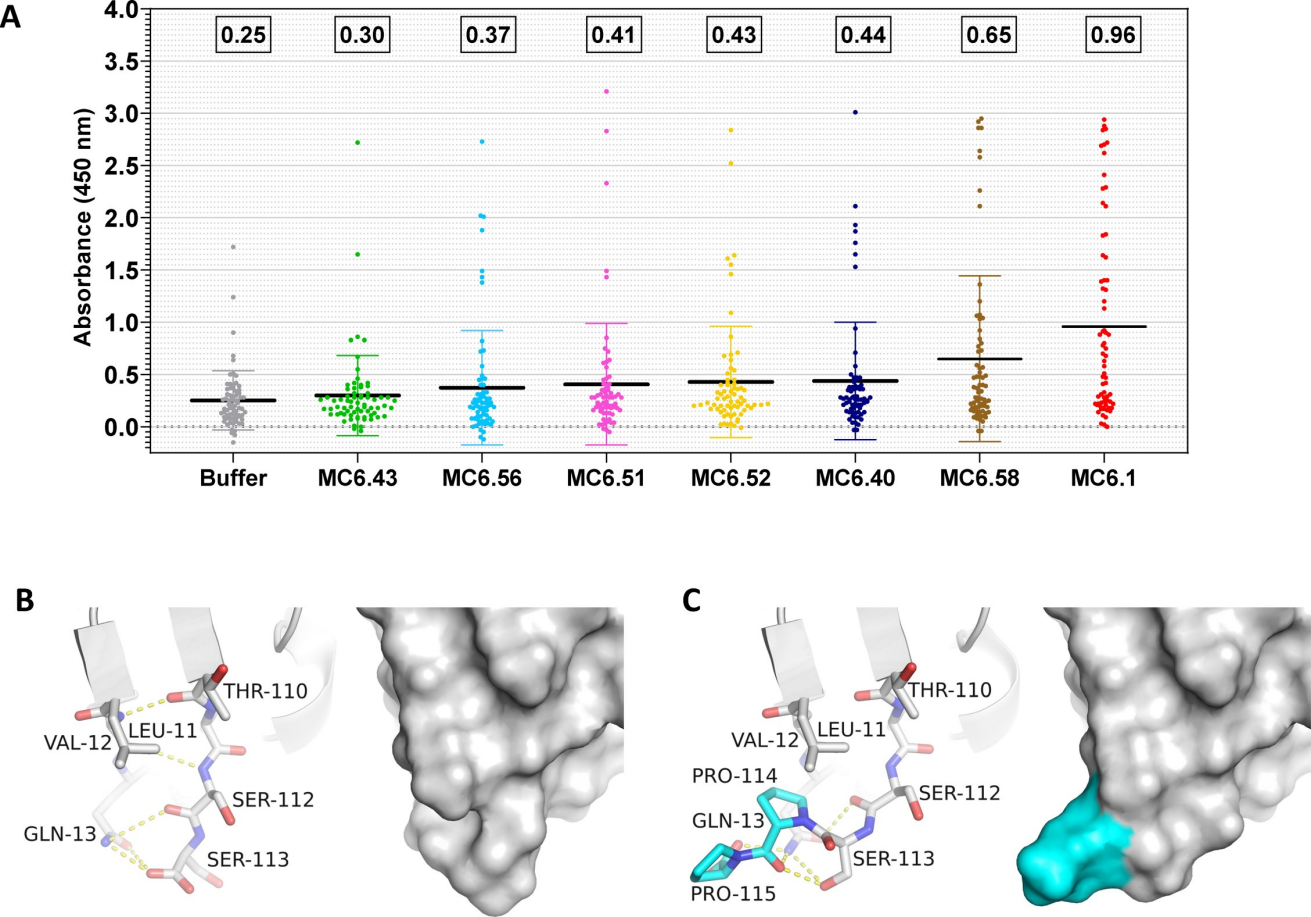
Comparison of pre-existing ADA reactivity of the unmodified VHH MC6.1 and six VHH variants. (A) ELISA absorbance values (X-axis) for each of the 70 individual sera is plotted for each respective VHH variant. ELISA absorbance signals were used to quantitate and compare pre-existing ADA binding. Mean and standard deviation are shown for each dataset, and relative mean values are indicated on top of each dataset. The unmodified VHH MC6.1 exhibits approximately a 4-fold higher mean signal as compared to buffer, while the control VHH MC6.40 as well as MC6.51, MC6.52, and MC6.56 exhibit approximately 1.5-fold higher mean signal. MC6.43 has quantitative pre-existing ADA binding comparable to the buffer control (~1.2), indicating that most pre-existing ADA reactivity has been successfully eliminated by the addition of two extra proline residues at the VHH C-terminus. (B) Structure model of the C-terminal portion of the unmodified VHH MC6.1 in cartoon and surface presentation compared to (C) MC6.43 with the pre-existing ADA design addition of amino acids Pro114 and Pro115 (cyan).

To compare pre-existing ADA reactivity across the various VHH variants, for each molecule the values of absorbance measured in response to the serum of each of the 70 donors was plotted, and the mean and standard deviation was calculated (Fig 6A). As expected, the unmodified MC6.1 had the highest level of pre-existing ADA reactivity. MC6.51, MC6.52 and MC6.56 had strongly reduced pre-existing ADA reactivities comparable to Cordy’s design [17] (MC6.40). MC6.43 was the most effective design with pre-existing ADA reactivity comparable to the buffer control, indicating that addition of prolines 114 and 115 efficiently abrogated pre-existing ADA binding. This can be explained by the significant structural differences the pre-existing ADA design imparted on the C-terminal VHH region. Structure models of the unmodified MC6.1 VHH (Fig 6B) and the MC6.43 variant (Fig 6C) highlight how the addition of the extra two proline residues reshapes the molecular surface at the C-terminus. Proline 114 hydrophobically stacks against Leu11, the main chain of Val12 and the side chain of Gln13 (Fig 6C). Numerous hydrogen bonds to the side chain of Gln13 stabilize the structural conformation and may contribute to both thermal and serum stability. Pre-existing ADA, like antibodies in general, recognize both conformational as well as linear epitopes. By reshaping the molecular surface of the C-terminal VHH epitope of MC6.43, it is possible to efficiently abrogate pre-existing ADA reactivity.

## Conclusion

Protein therapeutics, particularly engineered molecules, bear an inherent immunogenicity risk with potential impact on PK, safety and efficacy [5]. Pre-existing ADAs, in addition to treatment-induced anti-drug antibodies, are recognized as a development challenge for biotherapeutics [7–9]. Although some progress has been accomplished towards understanding origins and clinical impact of pre-existing ADA, much remains to be learned; e.g., understanding the specificity of pre-existing ADA is a pre-requisite to enable engineering strategies to abrogate pre-existing ADA binding and reduce potential impact on PK, efficacy, and safety [7, 8].

In this research, a structure-based approach was utilized to specifically engineer a known pre-existing ADA epitope at the C-terminus of isolated VH and VHH domains by leveraging 3D-structures to design modifications applicable to any VH(H), which should not impact developability or antigen binding. *In-silico* B cell epitope prediction algorithms were adopted for ranking of the modified VHH variants based on antigenicity; however, this analysis was insufficient in differentiating the parental from the engineered sequences. The limited applicability of *in-silico* immunogenicity predictions for pre-existing ADA reactivity required an experimental evaluation of numerous VHH variants, in which several designs were identified to have greatly reduced pre-existing ADA binding. Of particular note, the addition of two proline residues at the VHH C-terminus possessed no detectable pre-existing ADA binding resulting from its significant reshaped molecular surface and favorable developability characteristics. Thus, this particular modification may provide a broadly applicable solution to mitigate immunogenicity risk of VH(H)s and other antibody fragments in the clinic to increase safety and efficacy of this promising new class of biotherapeutics.

## Supporting information

S1 File (PDF)

S1 Raw images (PDF)
