# Supplementary material for: A structure-based engineering approach to abrogate pre-existing antibody binding to biotherapeutics
Source: PLoS One. 2021 Jul 23;16(7):e0254944. doi: 10.1371/journal.pone.0254944 (PMC8301669; doi:10.1371/journal.pone.0254944)

SUPPLEMENTAL ONLINE MATERIAL FOR

A structure-based engineering approach to abrogate pre-existing Ab binding to biotherapeutics

Joanne Lin, Stacey L. Lee, Anna M. Russell, Rong Fong Huang, Micheal A. Batt, Shawn S. Chang, Andrea Ferrante, Petra Verdino

Eli Lilly & Co, Lilly Biotechnology Center, San Diego, CA 92121, USA

Camelid VHH and autonomous human VH 3D X-ray structures and sequence data mining

The RCSB PDB data base (rcsb.org) was mined for camelid VHH and autonomous human VH 3D X-ray structures. Approximately 300 total PDB entries were retrieved for VHH or VH domains alone or in complex with other proteins. The largest number of VHH PDB entries were derived from llama (lama glama) (n=120), alpaca (Vicugna pacos) (n=82), and camel (Camelus dromedarius) (n=62). Fourteen structures were retrieved for autonomous human VH domains (refinement resolution range of 1.5 to 2.8Å). Due to their large number, VHH structures were divided into 3 subsets by species and only high-resolution structures with refinement resolutions better than 2Å were included for further analysis. This filtering strategy resulted in 47 structures for llama VHHs, 41 for alpaca VHHs, and 57 for camel VHHs. The program MOE [Molecular Operating Environment (MOE), 2019.01; Chemical Computing Group ULC] was used to remove non-VH(H) proteins, solvent atoms and ligands from the PDB files. VHH or VH CDRs were annotated according to Kabat's CDR definitions [Kabat, et al., 1971, Ann. NY Acad. Sci. 190:382-93] with CDRH3 in red, CDRH1 and CDRH2 in orange, frameworks in green). CDR regions, as expected, displayed high sequence and structural variability, while superimpositions including all framework residues yielded RMSD values well below 1Å indicating remarkable structural conservation.

S1 Fig. Llama (Lama glama) VHH amino acid sequences with refinement resolution range <2 Å (47 sequences) retrieved from PDB data base.

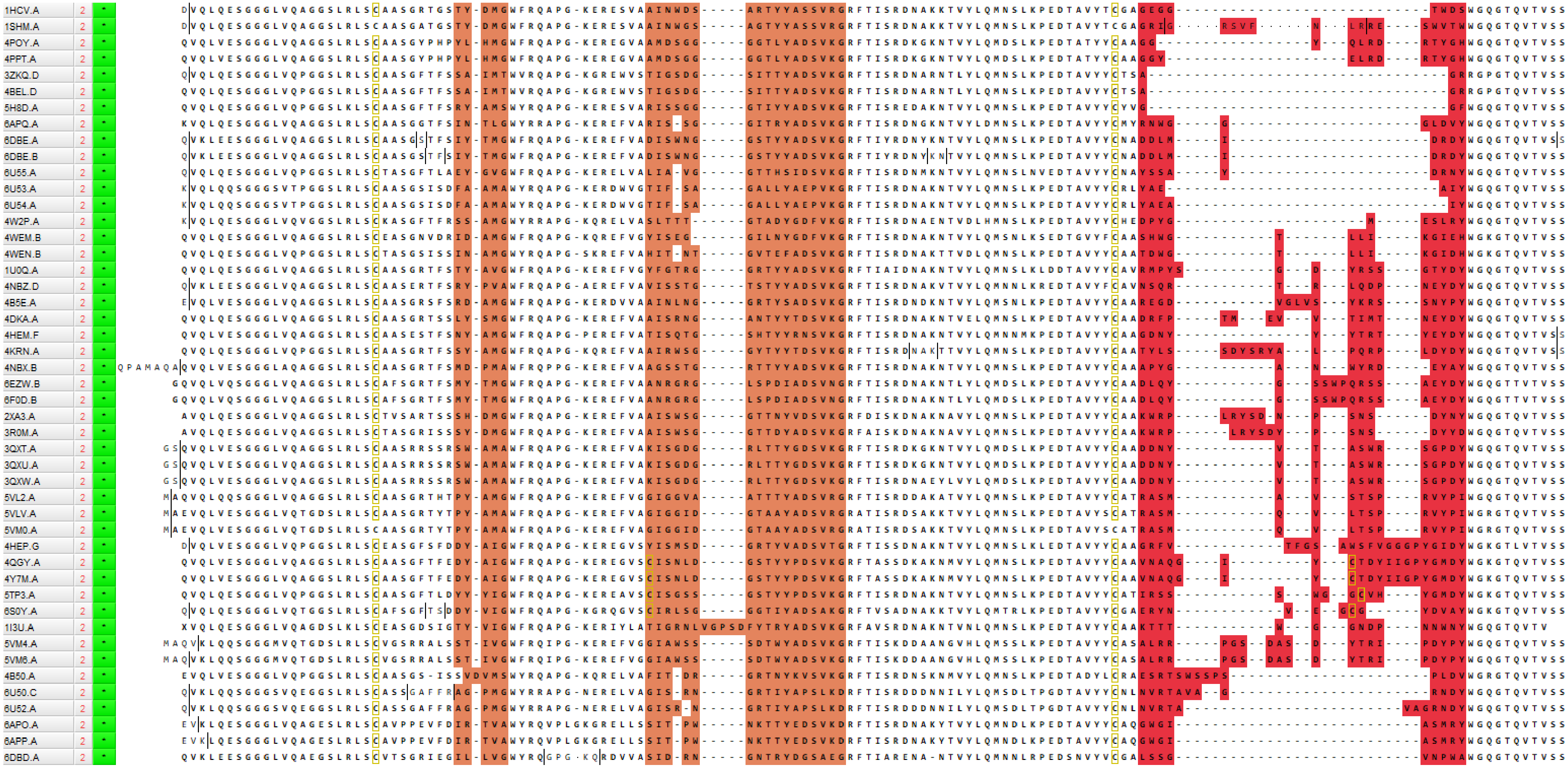

S2 Fig. Alpaca (Vicugna pacos) VHH amino acid sequences with refinement resolution range <2 Å (41 sequences) retrieved from PDB data base.

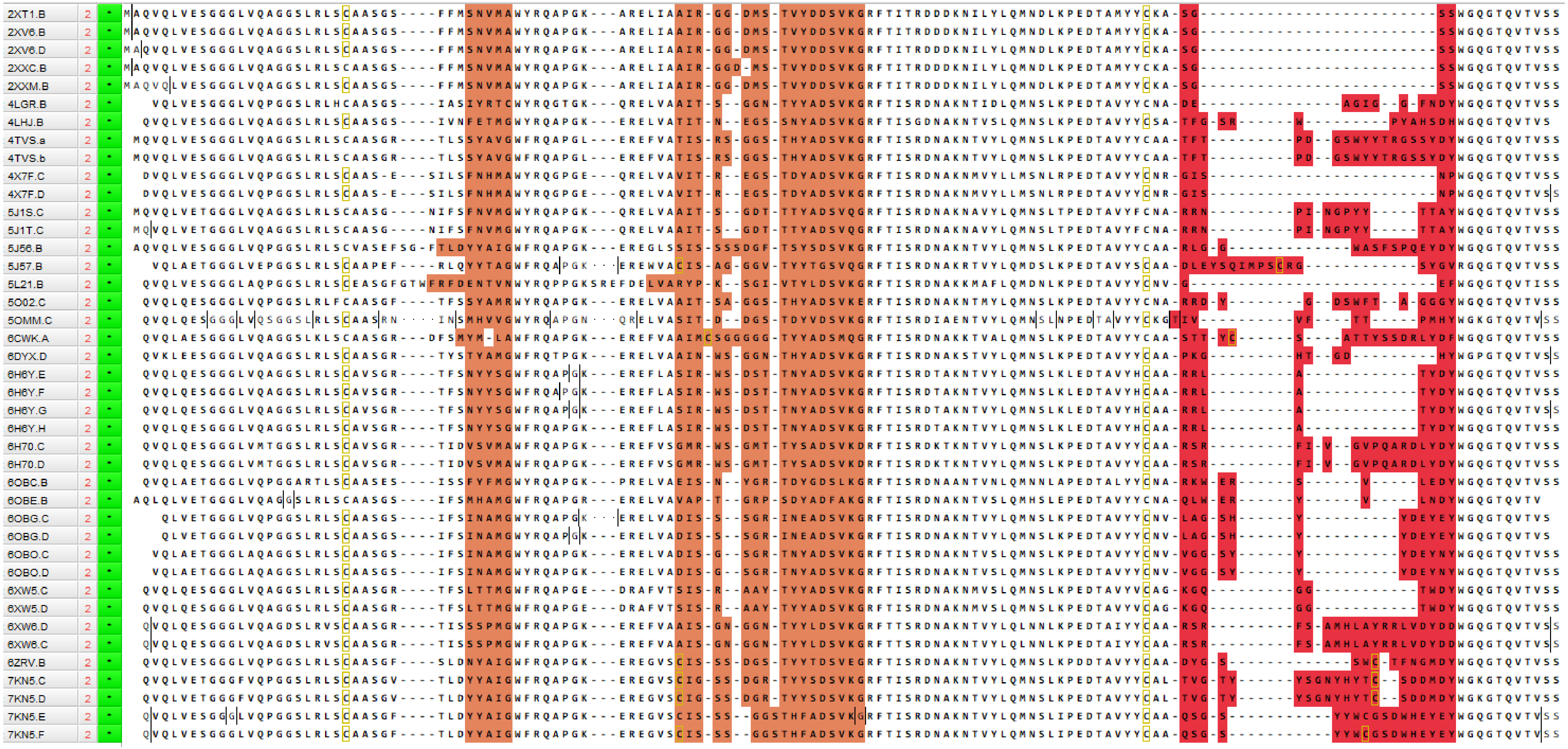

S3 Fig. Camel (Camelus dromedarius) VHH amino acid sequences with refinement resolution range <2 Å (57 sequences) retrieved from PDB data base.

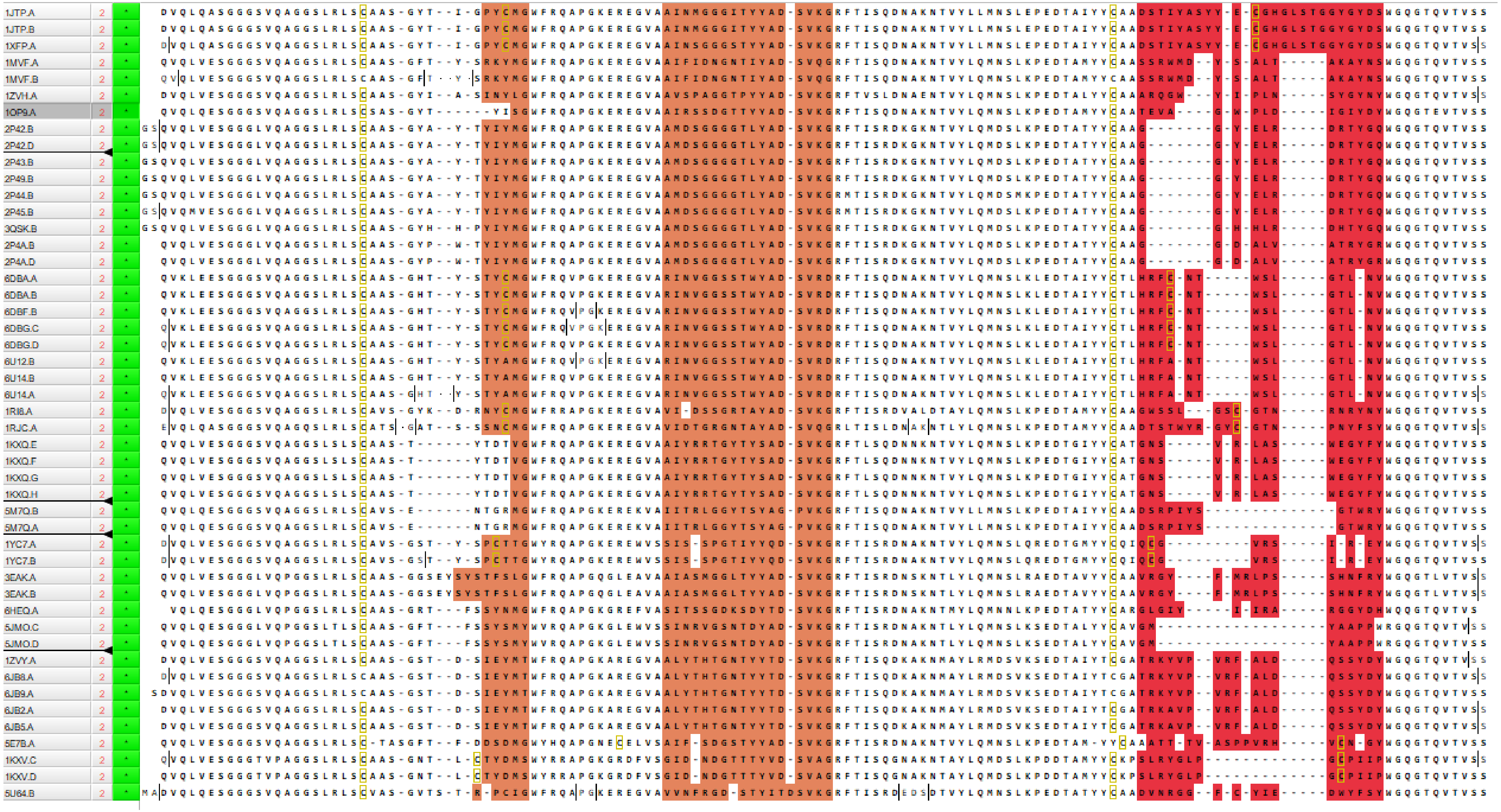

S4 Fig. Human (Homo sapiens) autonomous VH amino acid sequences with refinement resolution range <2.8 Å (14 sequences) retrieved from PDB data base

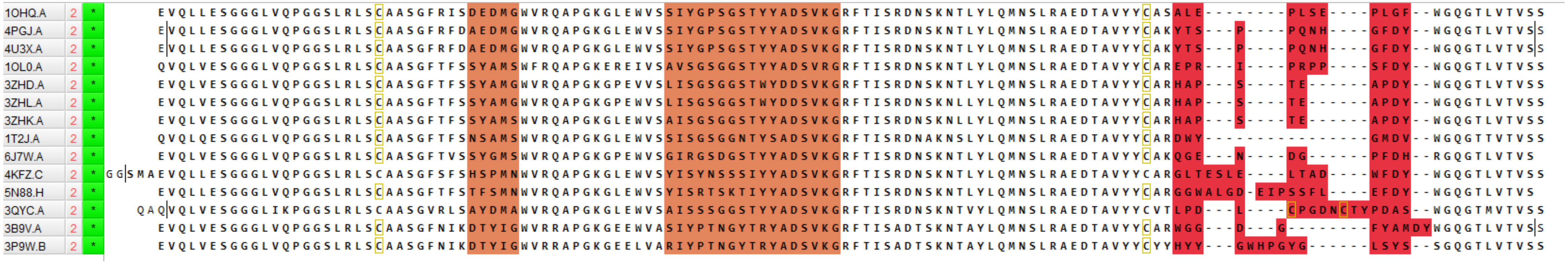

S5 Fig. Structure superimpositions of **camelid VHH and autonomous human VH 3D X-ray structures** retrieved from PDB data base.

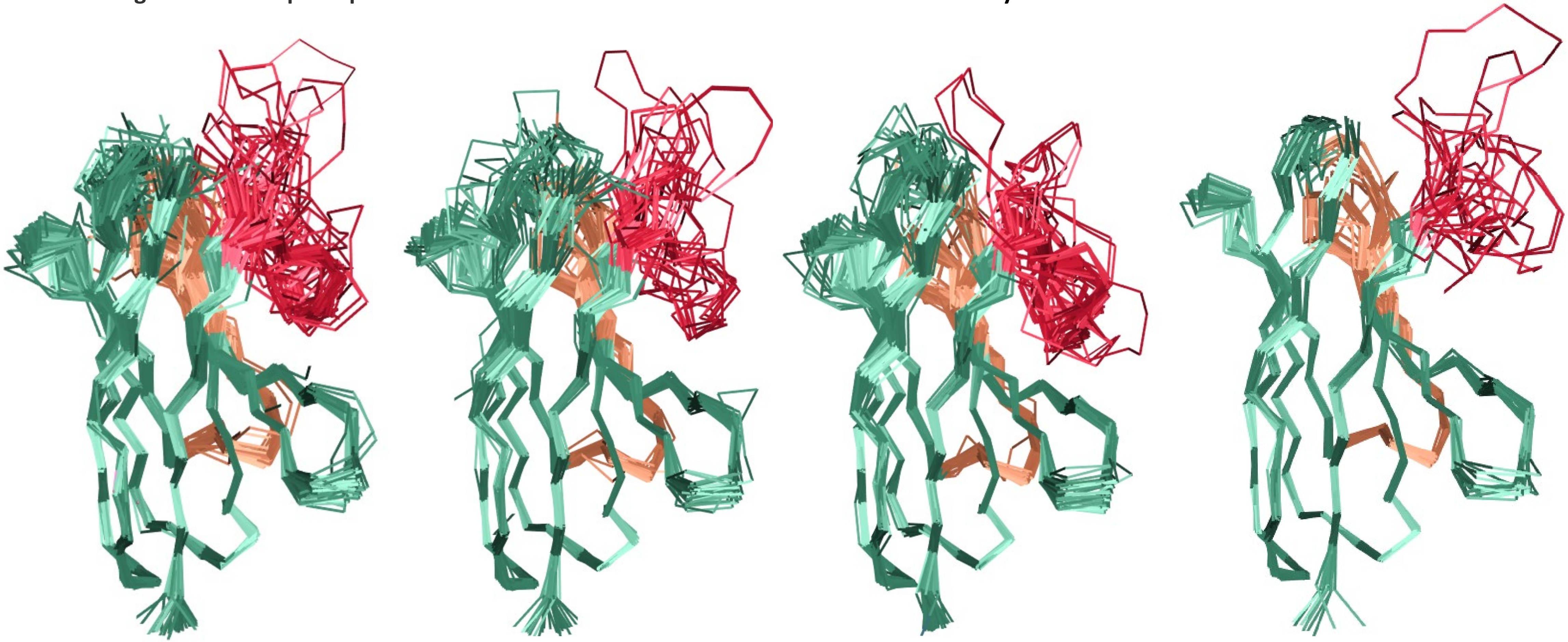

Framework superposed
Lama VHHs
(47 structures)
RMSD <0.5Å

Framework superposed
Alpaca VHHs
(41 structures)
RMSD <0.6Å

Framework superposed
Dromedary camel VHHs
(57 structures)
RMSD <0.6Å

Framework superposed
Human VHs
(14 structures)
RMSD <0.8Å

S6 Table. Final purification yields for the MC6.1 VHH with an unmodified C-terminus and 19 MC6.1 VHH variants with C-terminal designs from 100ml transiently transfected CHOK1 suspension cells.

VHH Variant	Purification Yield (mg)
MC6.1	6.5
MC6.40	6.6
MC6.41	5.8
MC6.42	4.3
MC6.43	6.4
MC6.44	6.4
MC6.45	5.7
MC6.46	6.0
MC6.47	5.2
MC6.48	5.7
MC6.49	8.0
MC6.50	8.0
MC6.51	7.2
MC6.52	7.5
MC6.53	7.2
MC6.54	8.9
MC6.55	4.1
MC6.56	8.1
MC6.57	7.6
MC6.58	7.0

S7 Fig. SPR sensorgrams used for global fitting analysis of the binding of MC6.1 VHH and selected VHH variants to their antigen (human serum albumin)

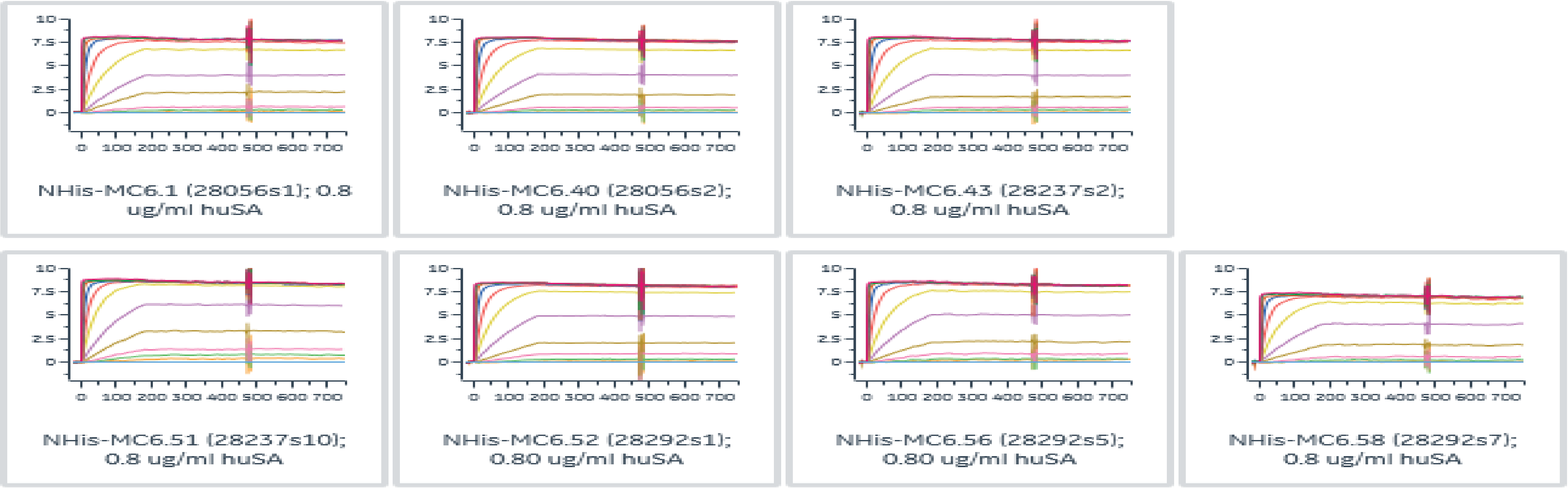

Supplement: S1 File — (PDF) [file pone.0254944.s001.pdf]
