# Supplementary material for: A structure-based engineering approach to abrogate pre-existing antibody binding to biotherapeutics
Source: PLoS One. 2021 Jul 23;16(7):e0254944. doi: 10.1371/journal.pone.0254944 (PMC8301669; doi:10.1371/journal.pone.0254944)

Lin et. al Figure 3A Raw data file – annotated

Images were acquired using a UVP EpiChemi3 Darkroom imager with white light and UVP Visionworks LS software. Images were saved as TIF files.

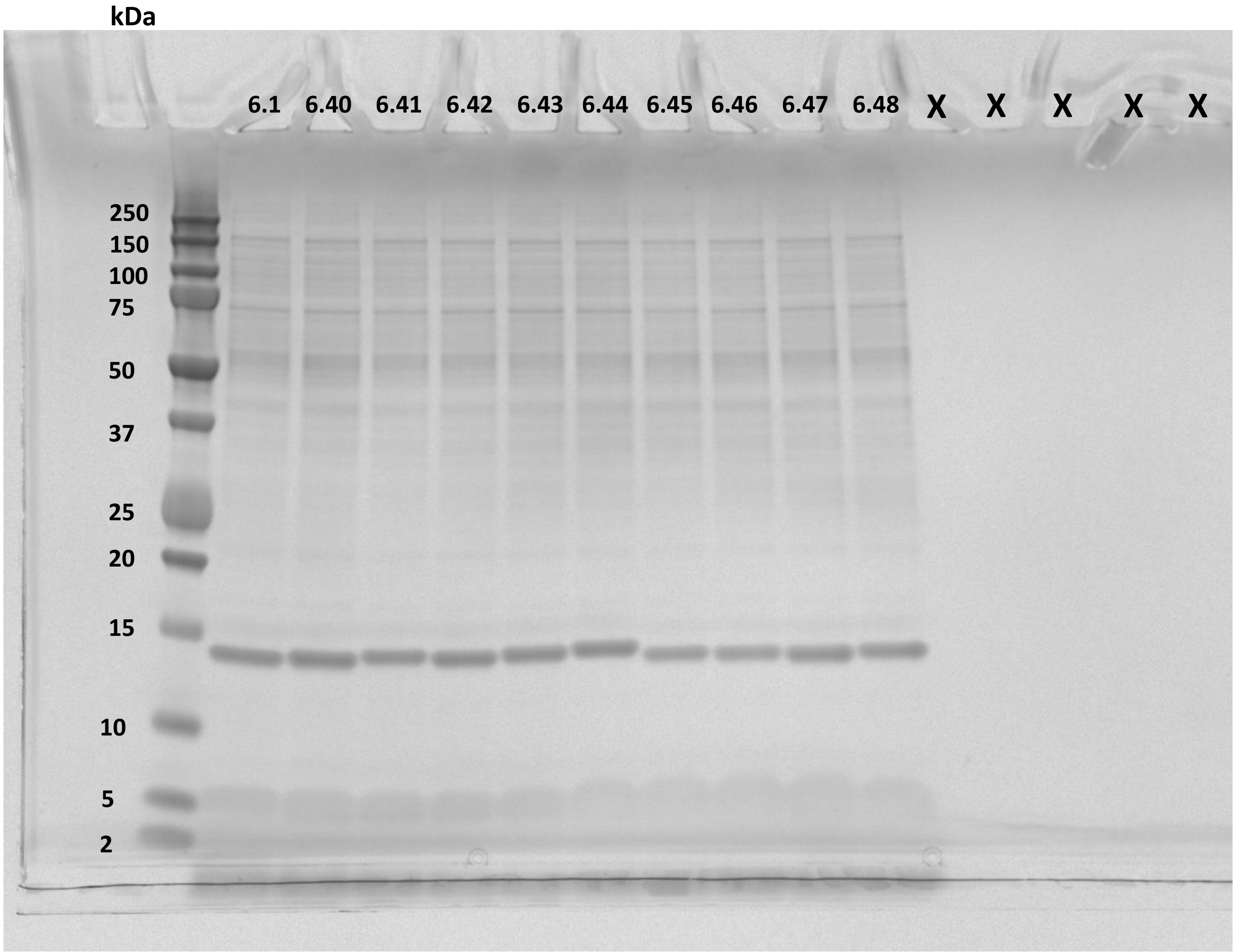

Lin et. al Figure 3A Raw data file – annotated

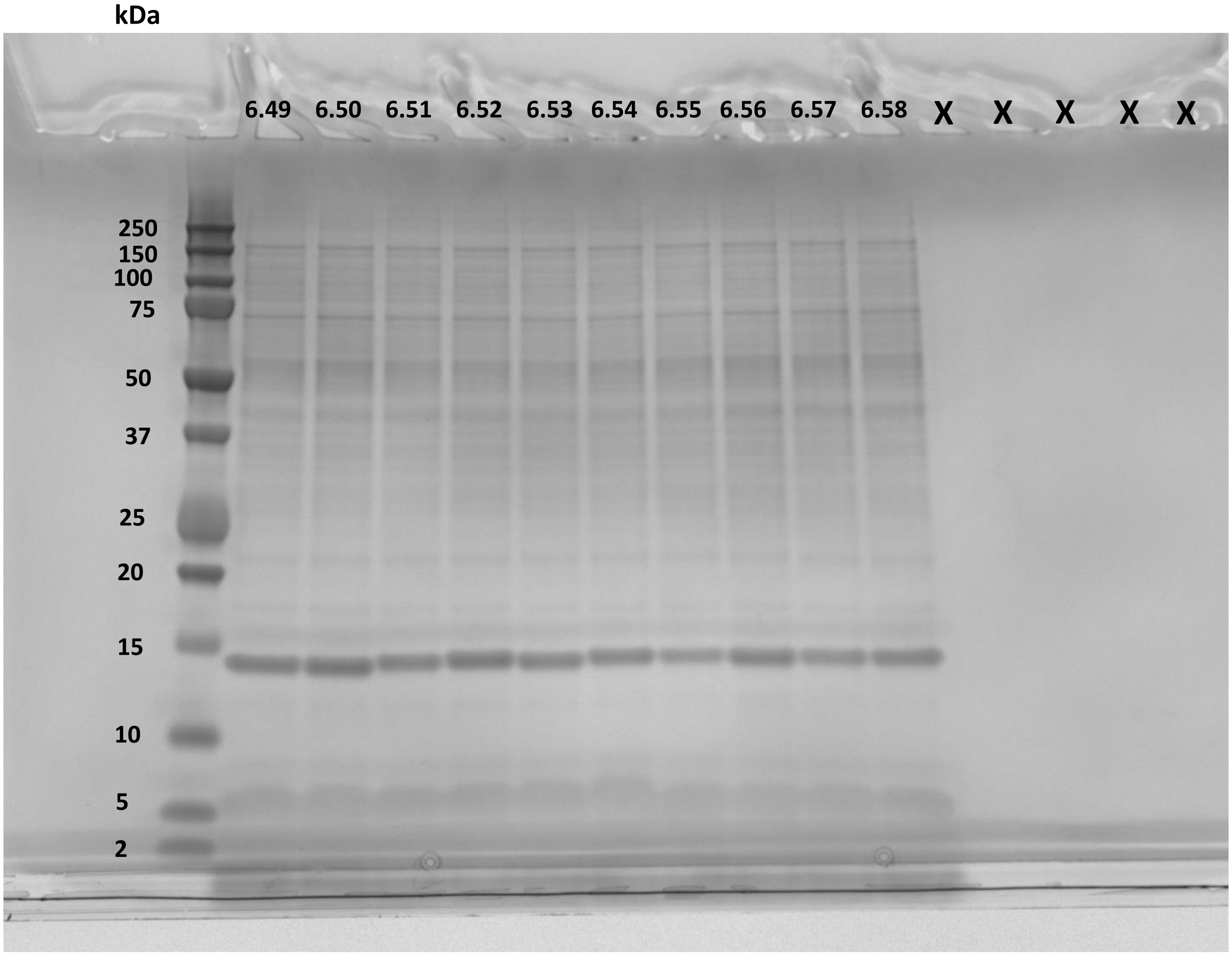

Supplement: S1 Raw images — (PDF) [file pone.0254944.s002.pdf]
